# The emerging role of ubiquitin-proteasome system dysfunction in the pathogenesis of perioperative neurocognitive disorders: a narrative review

**DOI:** 10.3389/fnbeh.2026.1762088

**Published:** 2026-02-12

**Authors:** Minghua Ma, Jing Yang, Yuan Yang, Lin Li

**Affiliations:** Department of Anaesthesiology, The First Affiliated Hospital, Harbin, Heilongjiang, China

**Keywords:** neuroinflammation, perioperative neurocognitive disorders, synaptic plasticity, ubiquitination, ubiquitin-proteasome system

## Abstract

Perioperative neurocognitive disorder (PND) is a prevalent and serious complication affecting the central nervous system following surgery, particularly among elderly patients. PND has a significant impact on patient prognosis and places a substantial burden on both individuals and the healthcare system. Despite its importance, the complex pathological mechanisms underlying PND remain inadequately understood, and there are currently no effective prevention or treatment strategies available. One critical factor contributing to PND is the imbalance in protein homeostasis, with the ubiquitin-proteasome system (UPS), recognized as the primary mechanism for protein quality control within cells. This review systematically discusses the crucial role of UPS dysfunction in the development of PND. Additionally, it analyzes potential biomarkers for diagnosing PND and explores treatment strategies targeting the UPS. This provides a new perspective for a deeper understanding of the molecular mechanisms involved in PND and lays a theoretical foundation for the development of new intervention methods.

## 1 Introduction

Perioperative neurocognitive disorders (PND) are prevalent complications of the central nervous system in elderly patients during the perioperative period. These disorders primarily present as either reversible or persistent impairments in cognitive functions, including memory decline, inattention, reduced learning ability, and executive function impairment. PND encompasses several conditions, including preoperative cognitive impairment, postoperative delirium (POD), delayed neurocognitive recovery (DNR), and postoperative cognitive dysfunction (POCD) (Evered et al., 2018). PND not only prolongs hospital stays and raises medical expenses, but it is also closely associated with long-term cognitive impairment and a heightened risk of dementia. As the global aging process accelerates, the burden of PND on the medical system is becoming increasingly significant (Cheng et al., 2022). However, the pathogenesis of PND is not yet fully understood, and the lack of effective intervention measures poses a significant challenge in perioperative management (Mao et al., 2025).

The ubiquitin-proteasome system (UPS) is a fundamental pathway in eukaryotic cells that selectively degrades misfolded, damaged, or short-lived regulatory proteins. This system consists of a cascade of reactions involving ubiquitin-activating enzymes (E1), conjugating enzymes (E2), and ligases (E3). The precise regulation of the UPS is essential for maintaining the integrity of the intracellular proteome (Rai et al., 2025). In the central nervous system, UPS not only participates in synaptic plasticity and memory formation (Rai et al., 2025), but also degrades intrinsically disordered proteins (IDPs) through the 20S proteasome in a non-ATP-dependent manner to prevent their abnormal aggregation (Riutin et al., 2024; Salcedo-Tacuma et al., 2025). A significant body of evidence suggests that UPS dysfunction is a fundamental pathological feature of several central nervous system diseases, including Alzheimer’s disease (AD) and Parkinson’s disease (PD) (Liang et al., 2023; Liu et al., 2021). UPS dysfunction can result in the abnormal aggregation of toxic proteins like tau and Aβ. It also disrupts mitochondrial homeostasis and organelle dynamics, affecting the localization and turnover of synaptic proteins. These changes contribute to the onset and progression of central nervous system diseases (Hegde et al., 2025; Zheng et al., 2016).

Anesthetics and surgical trauma are significant cellular stressors that can directly or indirectly disrupt various cellular protection mechanisms, including the UPS. An increasing number of preclinical and clinical studies indicate that dysregulation of the UPS may serve as a crucial link between perioperative stress and neuro-pathological damage in PND. Anesthesia and surgical trauma can notably impair UPS function, contributing to the onset and progression of PND through several core mechanisms: promoting abnormal protein aggregation, exacerbating neuroinflammation, damaging synaptic plasticity, and disrupting mitochondrial homeostasis. This article aims to systematically explore how anesthesia and surgery interfere with the UPS, the fundamental molecular mechanisms behind UPS dysregulation in PND, the advancements in related biomarker research, and potential prevention and treatment strategies targeting the UPS. This will provide a scientific perspective for a deeper understanding of PND and the development of new intervention targets.

## 2 UPS regulatory network

**FIGURE 1:**
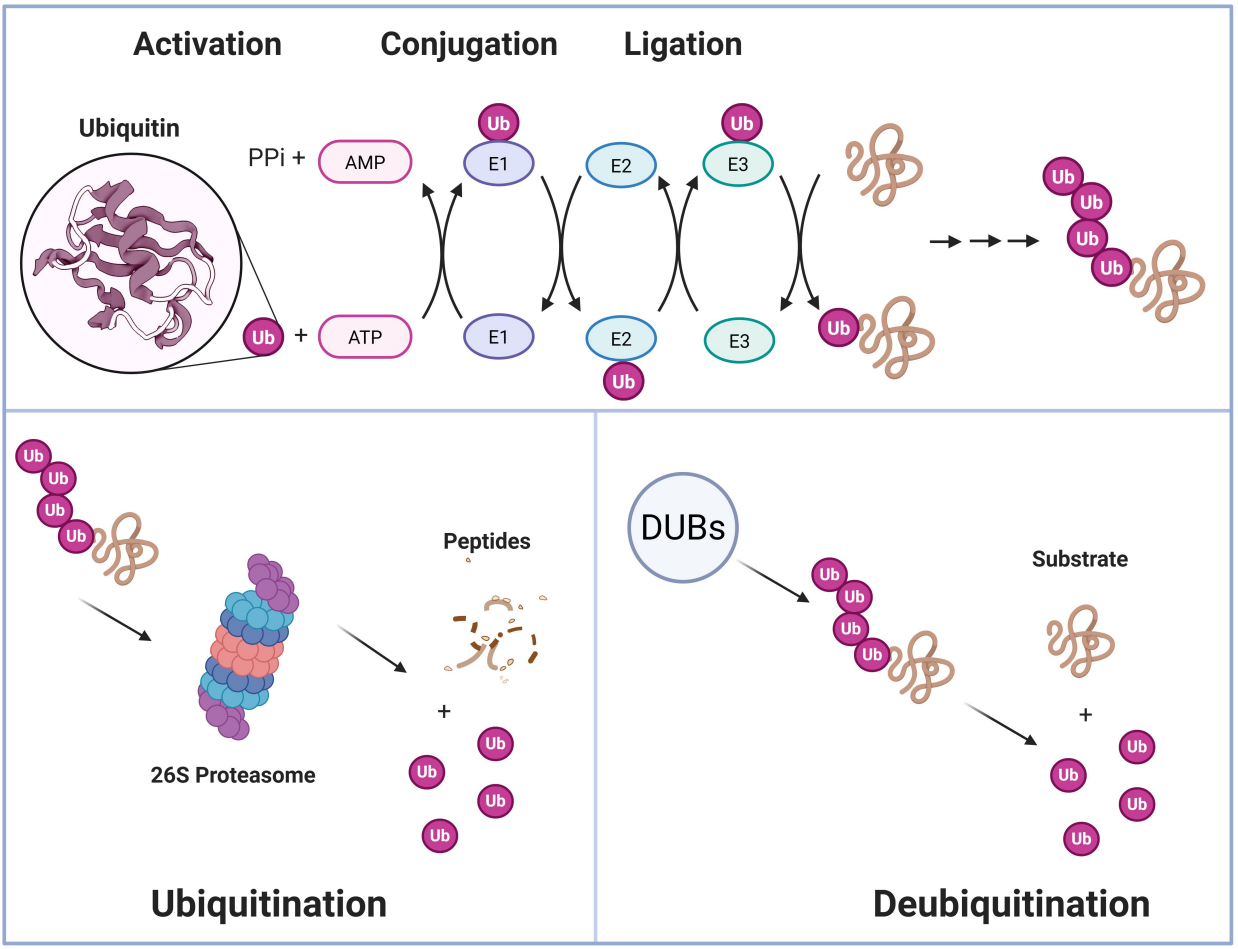
The ubiquitin-proteasome system (UPS) regulatory network. Ubiquitin first binds to a cysteine active site of an enzyme called ubiquitin-activating enzyme (E1 subunit) through an ATP-dependent reaction. Subsequently, the activated ubiquitin is transferred to the cysteine residue of the ligase (E2 subunit). Finally, through a specific E3 subunit, the E2 subunit can transfer polyubiquitin to the lysine residues of the substrate. Among them, the E3 ligase determines specificity, and K48 and K63 polyubiquitin chains are, respectively directed to the proteasome for degradation or non-degradation functions. DUBs can reversibly edit the ubiquitin chain, forming a bidirectional dynamic regulation; the 26S proteasome transports the substrate through 19S recognition, unfolding, and translocation to the 20S catalytic cavity for degradation.

The UPS is the main mechanism that regulates protein homeostasis in eukaryotic cells (Figure 1). Its primary function is to tag target proteins with covalently attached ubiquitin (Ub) molecules. These tagged proteins are then directed to the 26S proteasome for efficient and irreversible degradation (Kleiger and Mayor, 2014). This system is responsible for eliminating misfolded, damaged, or aged proteins within the cell to maintain proteostasis. Additionally, it plays a crucial role in precisely regulating various critical cellular processes, including cell cycle progression, DNA damage repair, signal transduction, and immune response (Amm et al., 2014).

The UPS consists of a cascade reaction involving ubiquitin-activating enzymes (E1), ubiquitin-conjugating enzymes (E2), and ubiquitin ligases (E3). E1 activates ubiquitin molecules in an ATP-dependent manner, forming a high-energy thioester bond with ubiquitin to create the E1Ub complex through a trans-thioesterification reaction. E3 specifically recognizes substrate proteins and catalyzes the formation of polyubiquitin chains linked through K48 or K63 lysine residues. More and more studies have confirmed that the alteration of E3 ubiquitin ligase levels after anesthesia and surgery plays a significant role in the development of PND (Table 1). K48-linked ubiquitin chains primarily mediate proteasomal degradation, while K63 chains are involved in non-degradative functions such as signal transduction (Brillada and Trujillo, 2022; Cheng et al., 2025; El-Hamaky et al., 2025). Additionally, deubiquitinating enzymes (DUBs) can reverse or remove ubiquitin chains, creating a dynamic bidirectional regulatory network (Hitora and Tsukamoto, 2025; Li S. et al., 2023). The 26S proteasome, as the degradation machinery of the UPS, is composed of a 20S core particle (CP) and a 19S regulatory particle (RP) (Brockmann et al., 2023; Ragwan et al., 2024). The 19S RP identifies polyubiquitinated proteins via ubiquitin receptors. It then employs ATPase subunits (Rpt1-6) to unfold the substrate and transport it into the catalytic chamber of the 20S CP for degradation (Lee et al., 2023; Ragwan et al., 2024). Notably, some disordered proteins can be directly degraded by the 20S proteasome without ubiquitination and 19S RP, and this non-classical pathway has special significance in neurodegenerative diseases (Oliveri et al., 2023; Sadahiro et al., 2024). In summary, the core regulatory nodes of the UPS involve the specific recognition and activity control of E3 ligases, the editing and reversal of ubiquitin signals by DUBs, and the adaptability of the proteasome. Moreover, the system can establish more complex regulatory hierarchies through cross-talk with other post-translational modifications such as SUMOylation. This multi-level regulation ensures that the UPS can accurately control the abundance and function of specific proteins in response to changes in the cell’s internal and external environment (Brillada and Trujillo, 2022; Jiang L. et al., 2025). In-depth research on the mechanism and regulation of the UPS enhances our understanding of fundamental cell biology and identifies crucial targets for addressing the pathogenesis and treatment strategies of various diseases, including cancer, neurodegenerative diseases, inflammation, and infectious diseases.

**TABLE 1:** The E3 ligases related to the perioperative neurocognitive disorders (PND) progression and their targets.

E3 ligases	Expression levels	Targets	Effects	References
MIB2	Upregulated in hippocampal neurons of mice exposed to sevoflurane	GPX4	Enhances the degradation of GPX4 by elevating its ubiquitination levels, which leads to neuronal ferroptosis.	Zhao et al., 2021
CHIP	Decreased in hippocampus of mice exposed to isoflurane	Synaptic protein	Decreases expression level of synapsin I and phosphorylation level of synapsin IS9, S427, and S605.	Xu et al., 2022
		HACE1	Increased in A549 and H1299 cell linesexposed to propofol	Li et al., 2020
Parkin	Increased in hippocampus of mice underwent exploratory laparotomy	mitochondrial-related proteins	Impairs mitochondrial autophagy by modifying mitochondrial-related proteins through ubiquitination.	Zhang et al., 2025a
TNFAIP1	Increased in hippocampus of mice underwent Aseptic laparotomy	SNAP25	Elevated levels of SNAP25 ubiquitination, resulting in damage to neuronal mitochondrial quality control.	Wang et al., 2023

## 3 The impact of anesthesia or surgical trauma on the UPS

### 3.1 The interference of anesthetics on the UPS function

In recent years, studies have shown that various commonly used anesthetics can directly disrupt the function of the UPS or influence the expression of its key proteins, resulting in either harmful or protective effects. Han et al. (2024) discovered that sevoflurane promotes UPS activation, which in turn increases the degradation of postsynaptic density region 95 protein (PSD-95), ultimately leading to cognitive impairment in young mice (Lu et al., 2017). Mind bomb-2 (MIB2) is a type of E3 ubiquitin ligase. Sevoflurane anesthesia has been shown to increase the expression of MIB2 in the hippocampal neurons of aged mice. MIB2 enhances the degradation of GPX4 by elevating its ubiquitination levels, which leads to neuronal ferroptosis and PND (Zhao et al., 2021). Ubiquitin-specific protease 7 (USP7) is an important member of DUBs. Xu et al. (2025) found that sevoflurane can affect the stability of PTEN by reducing the expression level of USP7, thereby reducing the ferroptosis of cardiomyocytes. Another study found that sevoflurane could inhibit the malignant progression of colorectal cancer by inhibiting the expression of circSKA3 and promoting the ubiquitination and degradation of β-catenin (Song et al., 2024). Isoflurane has been shown to contribute to the development of PND by impacting the function of the UPS. In aged mice exposed to isoflurane, there was a reduction in the expression of the ubiquitin E3 ligase protein carboxyl-terminus of Hsc70-interacting protein (CHIP), leading to UPS dysfunction. This may play a significant role in synaptic degeneration associated with isoflurane-induced PND (Xu et al., 2022). In addition, other intravenous anesthetics may also impact UPS function. For instance, previous studies have shown that propofol increases the expression of HECT domain and ankyrin repeat containing E3 ubiquitin protein ligase 1 (HACE1) and raises the ubiquitination level of optineurin. Furthermore, activating autophagy inhibited the proliferation of the lung cancer cell line A549 (Li et al., 2020). Zhang et al. (2021) found that Dexmedetomidine can increase the ubiquitination level of NLRP3 in microglia and promote its degradation, which is closely related to the effect of dexmedetomidine on. Anesthetics have a multifaceted impact on the function of the UPS. They can play a protective role by promoting the degradation of certain proteins while simultaneously contributing to pathological processes by reducing the levels of protective proteins. This offers a new perspective on how anesthetics interfere with neural function through the UPS.

### 3.2 Oxidative stress induced by surgical trauma leads to UPS dysregulation

Oxidative stress resulting from surgical trauma significantly contributes to the onset and progression of PND. The UPS serves as a crucial regulatory mechanism for cells to manage oxidative stress. Following anesthesia and surgery, a substantial amount of ROS is generated throughout the body, particularly in the central nervous system, surpassing the capacity of endogenous antioxidant defenses. These ROS can directly impair the UPS by oxidizing and modifying its essential components. Research indicates that ROS elevate ubiquitination levels by inhibiting deubiquitinating enzymes, rather than directly affecting proteasome activity (Kahles et al., 2021). The Keap1-Nrf2 pathway serves as the primary regulator of the antioxidant defense system. Research indicates that excessive ROS production in the hippocampus following surgery can disrupt the Nrf2/Keap1 pathway. This disruption results in the failure to activate the antioxidant defense system, which in turn exacerbates oxidative stress and neuroinflammation (Li L. et al., 2023). This oxidative microenvironment can directly harm proteasome function, leading to down-regulation of proteasome subunit transcription and disruption of the Nrf1-mediated regulatory pathway. This creates a vicious cycle of proteotoxic stress and increased neuronal vulnerability (Jiang S. et al., 2025). At the same time, ROS generated by mitochondria can disrupt the ubiquitin-proteasome regulatory mechanism of the TOM complex. This interference affects mitochondrial protein import and worsens the vicious cycle of oxidative stress and UPS dysfunction (Callegari, 2025; Liu F. et al., 2025). On the other hand, oxidative stress can disrupt endoplasmic reticulum homeostasis and indirectly impact UPS function by altering Ca^2+^ signaling pathways. This impairment of UPS function can further worsen proteotoxic stress, creating a vicious cycle of bidirectional interference (Kandel et al., 2024; Lee and Hong, 2022).

### 3.3 Blood-brain barrier injury and peripheral inflammation lead to UPS disruption

The systemic inflammatory response caused by surgical trauma can disrupt the blood-brain barrier (BBB) (Cao et al., 2018; Li et al., 2025a), allowing peripheral inflammatory factors to enter the central nervous system and leading to UPS dysfunction through multiple mechanisms. Cell damage following aseptic surgery can lead to a significant release of various damage-associated molecular patterns (DAMPs). These DAMPs can activate peripheral immune cells via multiple pattern recognition receptors, initiating systemic inflammatory responses (Alam et al., 2018; Maisat and Yuki, 2025; Venereau et al., 2012). Studies have shown that elevated levels of peripheral pro-inflammatory factors following surgery are the primary cause of disrupted integrity and increased permeability of the BBB. For example, abdominal surgery in mice can result in BBB damage and cognitive dysfunction in an IL-6-dependent manner (Hu et al., 2018). In addition, peripheral TNF-α can upregulate the expression of MMP-9 in brain cells (Ding et al., 2019). The abnormal increase of MMP-9 leads to the extensive hydrolysis of various tight junction proteins such as Claudin and Occludin, resulting in an increase in BBB permeability (Huang et al., 2022). When the BBB is damaged, various peripheral pro-inflammatory factors, DAMPs, and immune cells can enter the central nervous system freely. This influx further activates microglia and astrocytes, creating a vicious cycle of neuroinflammation.

Inflammatory markers at the molecular level can directly interfere with the function of the UPS. Research has shown that pro-inflammatory factors, such as IFN-γ, can alter the transcription and protein expression patterns of proteasome subunits in neurons. This alteration impairs proteasome function, reducing the efficiency of Aβ clearance and negatively impacting neurological function (Kandel et al., 2024; Woo et al., 2025). Aβ can degrade p-glycoprotein (P-gp) on the BBB by activating the ubiquitin-proteasome pathway. This creates a positive feedback loop that exacerbates UPS dysfunction (Vulin et al., 2023). Additionally, serum proteins like fibrinogen that leak from the periphery into the brain can interact with brain cells, leading to local inflammation and inhibiting proteasome activity (Roseborough et al., 2023). The relationship between peripheral inflammation and UPS dysfunction is also evident in the tryptophan metabolic pathway. The overexpression of indoleamine 2, 3-dioxygenase 1 (IDO1) in monocytes activates the kynurenine pathway, resulting in elevated levels of plasma kynuurine (Platten et al., 2019; Tian et al., 2025). After crossing the damaged blood-brain barrier, these metabolites are converted into neurotoxic substances like quinolinic acid within the central nervous system. This transformation contributes to the inhibition of UPS function and results in neurological impairment (Li et al., 2025b). Postoperative intestinal flora imbalance has been confirmed to be closely related to the occurrence of PND. The release of various enterogenic metabolites increases and they enter the brain through the BBB, which may promote UPS dysfunction and neuroinflammation (Han et al., 2024; Nargeh et al., 2021; Xiong et al., 2024). In summary, peripheral inflammation and BBB injury resulting from anesthesia and surgery contribute to UPS dysfunction via complex neuroimmune interactions. This UPS dysfunction, in turn, worsens neuroinflammation and disrupts protein homeostasis. Future research should focus on clarifying the impact of specific perioperative factors on UPS and exploring pharmacological interventions to maintain protein homeostasis and alleviate PND.

### 3.4 Glymphatic system impairment after anesthesia and surgery lead to UPS dysfunction

The glymphatic system is essential for clearing metabolic wastes from the central nervous system (Ding et al., 2025). Emerging evidence suggests that glymphatic dysfunction, which can be induced by anesthesia and surgery through various mechanisms, may represent a potential pathogenic pathway for PND (Ren et al., 2021). Surgical ablation of deep cervical lymph nodes, for example, worsens spatial learning deficits in APP/PS1 transgenic mice (Wang et al., 2019). Exposure to sevoflurane impairs glymphatic function in juvenile rodents by promoting the depolarization of aquaporin-4 (AQP4) water channels. This disruption hinders the clearance of cerebral metabolic wastes (Wang S. et al., 2024). Glymphatic dysfunction is closely linked to impairments in the UPS. Compromised glymphatic clearance efficiency results in the accumulation of aberrant proteins, such as Aβ and tau, in the brain. These proteins are key triggers for UPS dysfunction (Jia et al., 2025; Lin et al., 2022). Furthermore, impaired glymphatic function can worsen UPS deficits through inflammatory cascades. Accumulated misfolded proteins activate the NLRP3 inflammasome in microglia, leading to the release of pro-inflammatory cytokines. These cytokines further inhibit UPS function by interfering with proteasomal assembly, thereby creating a self-reinforcing vicious cycle (Gu et al., 2022). Anesthesia and surgery may significantly impair glymphatic function and disrupt the balance of the UPS, which could be key mechanisms contributing to the occurrence of PND.

## 4 The core molecular mechanism of the UPS dysfunction in PND

In recent years, numerous studies have indicated that UPS dysfunction may significantly contribute to the onset and progression of PND. Within the central nervous system, UPS is essential for cognitive functions as it regulates protein turnover, removes misfolded and oxidized proteins, and maintains synaptic plasticity. When UPS malfunctions, it results in abnormal protein aggregation and disturbances in protein homeostasis, ultimately impairing cognitive functions through various pathways. This discussion will explore the role of UPS dysfunction in the mechanisms underlying PND, focusing on aspects such as neuroinflammation, damage to synaptic plasticity, pathological protein deposition, and mitochondrial dysfunction.

### 4.1 UPS dysfunction triggers pathological protein deposition

The deposition of pathological proteins, such as tau and Aβ, is a key mechanism driving the occurrence and progression of PND (Jing et al., 2025; Sim et al., 2025). As the main intracellular protein degradation pathway, the impairment of the UPS is closely related to the hyperphosphorylation of tau and the deposition of Aβ (Hsieh et al., 2024; Jiang S. et al., 2025). In the pathogenesis of PND, factors like neuroinflammation, oxidative stress, and metabolic disorders resulting from surgical trauma can disrupt the normal functioning of the UPS. This disruption may occur through direct inhibition of proteasome activity or through abnormalities in the ubiquitination process. As a result, the compromised proteasome fails to efficiently clear misfolded proteins, leading to the accumulation of neurotoxic proteins such as tau and Aβ at synaptic sites. This accumulation directly impacts synaptic efficacy and plasticity (Chocron et al., 2022; Ribeiro et al., 2023); on the other hand, abnormal protein aggregation further disrupts mitochondrial function, resulting in neuronal damage and cognitive decline (Liu F. et al., 2025). Animal experiments have confirmed that increasing proteasome activity can significantly decrease the accumulation of pathological proteins and enhance spatial learning and memory abilities in aged mice (Parker et al., 2025). The autophagy-lysosome pathway is the primary mechanism for clearing Aβ. However, dysfunction in the UPS can indirectly destabilize the enzymes involved in the generation of Aβ or the proteins responsible for its clearance, resulting in abnormal Aβ aggregation. Research indicates that dexmedetomidine can enhance PND in mice by modulating the autophagy-lysosome pathway. This suggests that the coordinated dysregulation of the two main protein quality control systems—the UPS and the autophagy-lysosome pathway may play a crucial role in the development of PND (Liu B. et al., 2022; Wang Z. et al., 2025). These findings provide a new perspective on how UPS dysfunction leads to cognitive decline through protein toxicity stress.

### 4.2 UPS dysfunction leads to synaptic plasticity impairment

Studies have shown that the UPS not only regulates protein degradation but also plays a key role in synaptic plasticity and memory formation (Patrick et al., 2023). During aging, the activity of 20S and 26S proteasomes in the brain, particularly in the hippocampus, decreases significantly. Additionally, Aβ can inhibit synaptic proteasome activity. This reduction in synaptic proteasome function contributes to the loss of dendritic spines in hippocampal neurons and results in memory impairment in mice (Parker et al., 2025; Ribeiro et al., 2023). In the PND model of aged mice, a decrease in the expression of glucose transporter 1 (GLUT1) in hippocampal astrocytes was observed. This reduction led to a decline in dendritic spine density and impaired synaptic plasticity (Li et al., 2025c). The normal functioning of the proteasome depends on an adequate supply of ATP. Consequently, an insufficient energy supply in the brain during the perioperative period may directly impair UPS function, potentially causing irreversible damage to synaptic plasticity. Furthermore, the UPS plays a crucial role in regulating synaptic structure and function by degrading specific synaptic proteins. Research has shown that the deubiquitinating enzyme USP47 stabilizes AMPAR at the synaptic site by preventing the ubiquitination and degradation of the AMPAR subunit GluA1, thereby ensuring the maintenance of excitatory synaptic transmission (Yang et al., 2025). In the PND model, perioperative stress may disrupt the function of this deubiquitinating enzyme or E3 ubiquitin ligase (Wang et al., 2023; Zhang et al., 2025a), leading to excessive degradation of key receptors such as AMPAR and directly weakening synaptic transmission efficiency. Another study discovered that postoperative mice exhibited abnormal glutamatergic neurotransmission in the medial prefrontal cortex and hippocampus. This was characterized by an increased frequency of mEPSCs and enhanced presynaptic glutamate release (Zhang et al., 2025c). The UPS serves as a vital protein quality control system, and its dysfunction can result in abnormal accumulation or depletion of presynaptic and postsynaptic protein components. This imbalance may worsen the synaptic functional disorders mentioned above (Tisdale et al., 2022; Wang Y. Z. et al., 2024). In summary, perioperative stress-induced UPS dysfunction can impair synaptic plasticity by disrupting synaptic protein homeostasis, damaging synaptic structure and function, and creating a vicious cycle with energy metabolism disorders. This ultimately promotes the development of PND.

### 4.3 UPS dysfunction exacerbates neuroinflammation

In recent years, studies have confirmed that neuroinflammation caused by surgical trauma is a key pathological mechanism of PND. The dysfunction of the UPS initiates and amplifies central neuroinflammation through various mechanisms, which synergistically worsens neuronal damage and cognitive decline (Liu et al., 2023). Firstly, perioperative stress-induced UPS dysfunction can directly result in the abnormal activation of peripheral inflammatory signaling pathways. This disruption inhibits the ubiquitination and degradation of IκB, allowing NF-κB to remain continuously activated and translocate to the nucleus. Consequently, this process initiates the transcription of various inflammatory factor genes, including TNF-α, IL-1β, and IL-6 (Joshi et al., 2021; Zhong et al., 2024). These inflammatory factors remain elevated in PND patients for an extended period following surgery. This prolonged elevation disrupts the integrity of the blood-brain barrier, allowing these factors to enter the brain and create a neuroinflammatory microenvironment. Microglia, the innate immune cells of the central nervous system, play a crucial role in the onset and progression of PND through neuroinflammation driven by abnormal activation (Zhang and Yin, 2023). Studies have found that the polarization of microglia toward the pro-inflammatory M1 phenotype following anesthesia and surgery is a key factor in promoting neuroinflammation. This polarization amplifies the neuroinflammatory response by releasing pro-inflammatory factors. Additionally, UPS regulatory abnormalities can facilitate M1 polarization of microglia through multiple pathways (Luo et al., 2021; Xu et al., 2023; Zhang Y. et al., 2024). Additionally, pathological protein deposition resulting from UPS dysfunction can function as a damage-associated molecular pattern molecule (DAMPs), which continuously activates microglia. This activation leads to the significant release of inflammatory factors and may mediate complement system-dependent synaptic phagocytosis, a critical mechanism behind synaptic loss in PND (Ribeiro et al., 2023; Tan et al., 2023; Zingale et al., 2025). Moreover, oxidative stress caused by UPS dysfunction significantly increases ROS levels. This rise in ROS can activate pattern recognition receptors, including the NLRP3 inflammasome in microglia, leading to the maturation and release of inflammatory factors like IL-1β (Zhang et al., 2021; Zhu et al., 2022). Elevated ROS and inflammatory factors further inhibit proteasome activity, forming a positive feedback loop and continuously amplifying neuroinflammation and oxidative damage (Chen et al., 2022; Chiang et al., 2024). In summary, UPS dysfunction worsens the cycle of perioperative neuroinflammation by directly activating inflammatory signals, causing pathological protein deposition and increasing oxidative stress. This cascade ultimately results in synaptic damage and neuronal dysfunction, which are critical factors in the development of PND. Interventions aimed at key points in the UPS-neuroinflammation axis could offer new approaches for preventing and treating PND.

### 4.4 UPS dysfunction and mitochondrial damage

**FIGURE 2:**
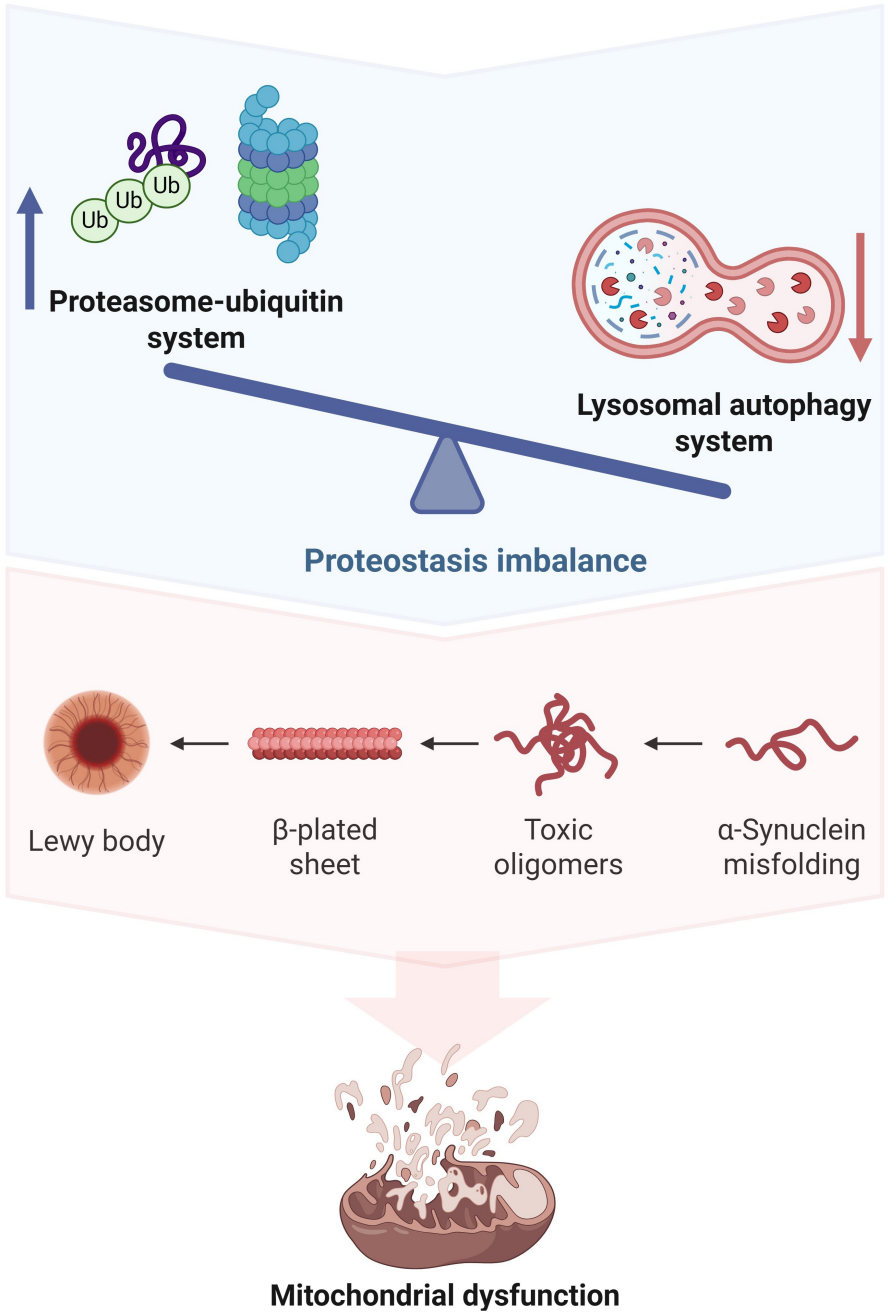
Ubiquitin-proteasome system (UPS) dysfunction triggers mitochondrial damage. UPS dysfunction and impaired autophagy lead to protein homeostasis imbalance, which can damage mitochondrial structure by selectively degrading mitochondrial membrane proteins. In addition, UPS dysfunction results in a decreased ability to clear misfolded proteins, thereby interfering with mitochondrial division, fusion, and autophagy functions and exacerbating mitochondrial oxidative stress. Therefore, UPS dysfunction plays a crucial role in mitochondrial dysfunction.

Recent studies have found that UPS dysfunction can cause mitochondrial damage through multiple pathways (Figure 2), thereby participating in the pathological process of PND (Liu F. et al., 2025; Zhang Z. et al., 2024). Abnormalities in the UPS system can result in the selective degradation of the outer mitochondrial membrane protein TOM40. This degradation leads to a decrease in mitochondrial membrane potential, accumulation and loss of mtDNA, and ultimately, mitochondrial dysfunction (Vasquez et al., 2024). Secondly, UPS dysfunction disrupts the dynamic balance of mitochondrial fission and fusion, affecting the stress response ability of mitochondria to protein toxicity (Oshima et al., 2021). Additionally, UPS dysfunction can worsen mitochondrial oxidative stress, leading to further damage to mitochondrial function due to an increase in the production of reactive oxygen species (Oshima et al., 2021). During the perioperative period, stress factors like surgery and anesthesia can disrupt UPS function, which may result in neuronal dysfunction and cognitive impairment (O’Gara et al., 2021). Parkin is a crucial E3 ubiquitin ligase. Research has shown that anesthesia and surgery can elevate Parkin expression in the hippocampus. This increase may impair mitochondrial autophagy by modifying mitochondrial-related proteins through ubiquitination, contributing to the development of PND in mice (Zhang et al., 2025a). Wang et al. discovered that the expression of TNF alpha induced protein 1 (TNFAIP1) in the hippocampus of mice increased following abdominal surgery. TNFAIP1 is a type of ubiquitin ligase, and its N-terminal 1-96 amino acid residues form a covalent bond with the SNAP25 protein. This interaction leads to elevated levels of SNAP25 ubiquitination, resulting in damage to neuronal mitochondrial quality control and the onset of PND (Wang et al., 2023). Mitochondrial damage resulting from UPS dysfunction plays a significant role in the mechanism behind PND occurrence.

## 5 Exploration of UPS-related components as potential biomarkers for PND

The diagnosis of PND currently relies primarily on neuropsychological tests, which are often subjective and time-consuming. Consequently, it is essential to identify objective and sensitive biomarkers for the early assessment of PND risk (Liu J. et al., 2025). Components related to UPS may reflect the pathological process of protein imbalance within nerve cells earlier than traditional markers of nerve damage, making them potential ideal biomarkers (Brown et al., 2018; Dulka et al., 2020). Components related to UPS may reflect the pathological process of protein imbalance within nerve cells earlier than traditional markers of nerve damage, making them potential ideal biomarkers (Zhang et al., 2025a). Mind bomb-2 (MIB2) is another E3 ubiquitin ligase, and sevoflurane anesthesia can increase the expression of MIB2 in the hippocampus of aged mice, thereby causing neuronal ferroptosis and the occurrence of PND (Zhao et al., 2021). Wang et al. (2023) found that after abdominal surgery, the expression of the ubiquitin ligase TNFAIP1 in the hippocampus of mice increased, resulting in a higher level of ubiquitination of SNAP25. These studies suggest that variations in the levels of ubiquitin ligases could serve as potential biomarkers for PND. Furthermore, in AD mouse models and investigations into age-related cognitive decline, researchers observed an abnormal accumulation of ubiquitinated proteins in the hippocampus, along with decreased proteasome activity. Moreover, there were age-dependent changes in proteasome activity and in the targeting of K48 polyubiquitinated proteins for degradation in the hippocampus of aged rats (Dulka et al., 2020; Hartz et al., 2018; Patrick et al., 2024). These findings provide a theoretical basis for exploring UPS markers in PND.

In recent years, research has increasingly focused on detectable UPS components in peripheral blood, paving the way for the development of non-invasive biomarkers. Notably, the study of circulating proteasomes has emerged as a significant area of interest. Research indicates that the activity of proteasomes in the peripheral blood of patients with mild cognitive impairment demonstrates a characteristic decrease (Yun et al., 2020). More importantly, the levels of ubiquitinated proteins and proteasome activity in the peripheral blood red blood cells of schizophrenia patients align with those found in brain tissue. This strongly demonstrates the feasibility of monitoring the UPS status of the central nervous system through peripheral blood (Bousman et al., 2019). These findings indicate that monitoring proteasome activity or the levels of ubiquitinated proteins in peripheral blood could serve as an effective method for assessing the risk of PND. Due to the complex pathological mechanisms underlying PND, a multi-marker detection strategy is anticipated to enhance early detection rates significantly. However, research linking UPS-related markers to PND is still in its early stages, and further studies are necessary to identify and evaluate these markers.

## 6 Potential intervention of targeting UPS malfunction for PND treatment

Given the crucial role of UPS dysfunction in PND, targeting UPS function may present a promising new strategy for both the prevention and treatment of PND (Table 2). Research indicates that dexmedetomidine can enhance ubiquitination, promoting the degradation of NLRP3 inflammasome-related proteins. This process inhibits postoperative neuroinflammation and improves cognitive function in mice (Zhang et al., 2021). UAF1 is a binding partner of the deubiquitinating enzyme UPS1. Zhu et al. (2022) found that the knockdown of UAF1 could promote UPS1-mediated NLRP3 ubiquitination and degradation, thereby alleviating sevoflurane-induced neuroinflammation and cognitive impairment in rats. Zhao et al. (2021) found that knocking down the E3 ubiquitin ligase MIB2 reduces the ubiquitination level of GPX4, inhibiting its degradation. This action alleviates sevoflurane-induced neuronal ferroptosis and cognitive impairment (Zhao et al., 2021). Moreover, it has been found that inhibiting the ubiquitin ligase TNFAIP1 can alleviate PND in mice by reducing neuronal pyroptosis (Wang et al., 2023). Recent structure-based virtual screening and biological validation have revealed that tamarixetin treatment enhances the ubiquitination of mitofusin 2, which helps alleviate PND by promoting mitophagy (Zhang et al., 2025b). These studies provide direct theoretical evidence for targeting UPS dysfunction to alleviate PND.

**TABLE 2:** Evidence for attenuation of perioperative neurocognitive disorders (PND) through modulation of ubiquitin-proteasome system (UPS) function.

Drugs	Targets	PND models	Experimental subject	Effects	References
Dexmedetomidine	NLRP3	Exploratory laparotomy	12 months male C57BL/6J mice and primary microglia	Attenuated the hippocampal brain inflammation by promoting NLRP3 inflammasome degradation via the autophagy-ubiquitin pathway.	Zhang et al., 2021
Tamarixetin	PINK1	Exploratory laparotomy	8∼12 weeks male C57BL/6J mice, HT22 and BV2 cells	Promotes PINK1 stabilization and strengthened PINK1-translocase of outer mitochondrial membrane 40 interactions, while facilitating Parkin recruitment to mitochondria and enhancing mitofusin 2 ubiquitination, ultimately promoting mitophagic flux in both neuron and microglia.	Zhang et al., 2025b
AAV-shUAF1	NLRP3	Sevoflurane exposure	Neonatal rats	Promotes the ubiquitination-mediated degradation of NLRP3 mediated by USPI, thereby inhibiting oxidative stress and inflammatory responses.	Zhu et al., 2022
AAV-shTNFAIP1	TNFAIP1	Aseptic laparotomy	12 months male C57BL/6J mice and HT22 cells	Inhibits the ubiquitination and degradation mediated by TNFAIP1 for SNAP25, thereby restoring autophagy function of neurons and alleviating neuronal pyroptosis.	Wang et al., 2023
si-MIB2	MIB2	Sevoflurane exposure	15 months male C57BL/6 mice	Reduces the ubiquitination level of GPX4 and inhibit its degradation, thereby alleviating sevoflurane-induced neuronal ferroptosis.	Zhao et al., 2021

Currently, there are few drugs that specifically target the UPS for the prevention and treatment of PND. However, inhibiting or activating the enzymes that regulate the UPS may present a significant opportunity for PND treatment. As a core component of the UPS, proteasome activators can counteract UPS dysfunction and facilitate the clearance of pathological proteins, thereby exerting neuroprotective effects. The activation of cAMP-dependent Protein Kinase A (PKA) and cGMP-dependent Protein Kinase G (PKG) enhances the activity of the 26S proteasome, promoting the degradation of misfolded proteins (Goldberg et al., 2021). Rolipram is a phosphodiesterase 4 (PDE4) inhibitor that increases intracellular cAMP levels by inhibiting PDE4. In an AD mouse model, rolipram treatment was shown to restore the reduced synaptic proteasome activity caused by Aβ and enhance the cognitive function of the mice (Ribeiro et al., 2023). Similarly, another PDE4 inhibitor, roflumilast, also improves memory impairment in AD mice by enhancing the cAMP/PKA signaling pathway (Ashour et al., 2021). Moreover, PDE5 inhibitors like sildenafil and tadalafil enhance PKG activity by inhibiting cGMP hydrolysis. This action helps reduce the levels of misfolded proteins in the brain by activating the proteasome (Moore et al., 2024; VerPlank et al., 2022). These findings indicate that activators of PKA or PKG could potentially alleviate PND by improving the function of the UPS.

Recent studies have shown that inhibiting the endogenous proteasome inhibitor USP14 can significantly reduce neuronal damage caused by Aβ oligomers, highlighting an important target for the development of new neuroprotective drugs (Kiprowska et al., 2017; Lee et al., 2018). The selective USP14 inhibitor b-AP15 has shown significant therapeutic effects in various neurodegenerative disease models, especially in the regulation of tau protein pathology (Li et al., 2024; Ming et al., 2022). These findings establish a theoretical foundation for developing small molecule drugs that target USP14. Additionally, E3 ubiquitin ligases represent significant targets for drug development. In models of Parkinson’s disease, regulating the mitochondrial E3 ligase MITOL/MARCH5 has been shown to improve mitochondrial defects in neurons, and inhibiting USP14 can further amplify this protective effect (Takeda et al., 2021). Moreover, the FBXL19 member of the SCF E3 ligase complex regulates cell migration through ubiquitination, providing a new target for the development of modulators for neuroinflammation (Do and Baek, 2021). The regulation of key enzymes in the UPS appears to be a crucial focus for developing drugs to treat PND.

Additionally, non-pharmacological interventions could significantly contribute to the prevention and treatment of PND by enhancing UPS function. For example, optimizing perioperative management can mitigate the effects of perioperative stressors on the UPS (O’Gara et al., 2021). Preoperative cognitive training and early postoperative cognitive rehabilitation exercises may partially compensate for the effects of UPS dysfunction by enhancing the intrinsic stress resistance and synaptic plasticity of neurons (Jiang et al., 2024; Liu Y. et al., 2022). Recent studies have focused on the influence of gut microbiota and their metabolites on the central nervous system via the gut-brain axis. Dietary restriction, as a potential intervention, may positively impact cognitive impairment by reducing neuroinflammation (Que et al., 2024; Ren et al., 2024; Wang H. et al., 2025). These studies all provide new ideas for developing PND prevention and treatment strategies based on UPS dysfunction.

## 7 Limitations and future directions

Although the role of UPS dysfunction in the pathogenesis of PND has gradually garnered attention, significant knowledge gaps persist in current research, limiting our comprehensive understanding of the pathological mechanisms underlying PND and the development of effective intervention strategies. Firstly, the molecular mechanisms by which UPS is implicated in PND remain insufficiently elucidated. Existing studies predominantly focus on neurodegenerative diseases, yet the specific factors that trigger PND and particularly disrupt UPS function remain unclear. Secondly, the interactions between UPS and other pathological systems in PND are largely unexplored. Finally, current intervention strategies are primarily tested in AD or aging models, while the acute and reversible characteristics of PND may necessitate distinct treatment windows and dosage regimens. Moreover, due to the scarcity of clinical samples from PND patients, the feasibility of applying UPS-related biomarkers to PND has not been thoroughly evaluated.

Consequently, future research should prioritize several key directions. Firstly, it is essential to deepen mechanistic investigations and employ multi-omics technologies to analyze how anesthetics and surgical stress directly target UPS components to facilitate the onset of PND. Additionally, targeted intervention strategies specific to PND that focus on UPS, including preclinical testing of small molecule activators, should be further developed. Future studies should design clinical trials to assess the safety and efficacy of these interventions during the perioperative period and examine the potential for combined therapies. Finally, based on the dynamic changes in UPS components, more specific biomarkers need to be identified in the blood or cerebrospinal fluid of PND patients. Therefore, research on UPS dysfunction in PND is still in its nascent stages. Integrating interdisciplinary approaches is necessary to bridge the gap between mechanistic insights and translational applications. These efforts are anticipated to pave the way for precise diagnosis and treatment of PND.

## 8 Conclusion

Perioperative neurocognitive disorder is a prevalent central nervous system complication in elderly patients after surgery, significantly worsening their long-term prognosis and placing a substantial burden on the medical system. The pathogenesis of PND is not yet fully understood and likely involves complex interactions among multiple factors, including neuroinflammation, neurotransmitter imbalances, epigenetic modifications, and disorders of the gut-brain axis. Consequently, there are currently no definitive biomarkers or specific prevention and treatment strategies available.

**FIGURE 3:**
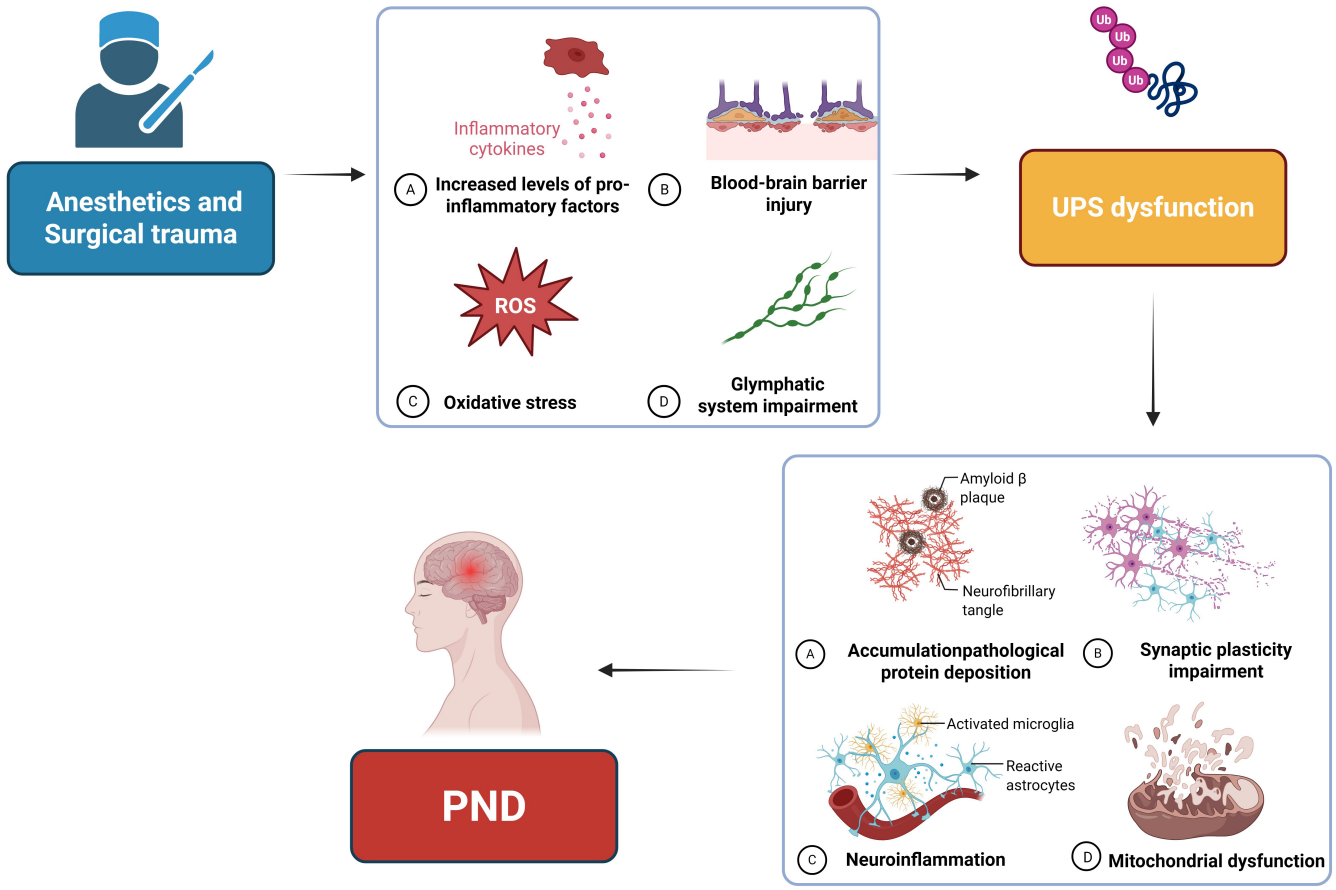
The mechanism by which anesthetics and surgical trauma promote perioperative neurocognitive disorders (PND) through triggering ubiquitin-proteasome system (UPS) dysfunction. This review highlights the critical role of UPS dysfunction, a novel and widespread mechanism in the development of PND. Perioperative stress from anesthesia and surgery, including anesthetic-induced inhibition, oxidative stress, and inflammatory factor activation, triggers UPS dysfunction in the central nervous system, particularly the hippocampus. This leads to accumulation of misfolded proteins like Aβ, driving a vicious cycle of neuroinflammation, synaptic damage, and mitochondrial dysfunction, ultimately resulting in neuronal and cognitive deficits.

The UPS is a fundamental regulatory system responsible for maintaining protein homeostasis in the central nervous system, and its dysfunction has garnered increasing attention in relation to PND in recent years. This review systematically highlights the critical role of UPS dysfunction as a novel and widespread mechanism underlying the occurrence and progression of PND (Figure 3). Existing evidence illustrates a causal chain linking perioperative stress to long-term cognitive impairment. The stress associated with anesthesia and surgery—characterized by the direct inhibition of anesthetic drugs, oxidative stress damage, and the remote regulation of inflammatory factors—serves as an initiating factor that triggers UPS dysfunction in the central nervous system, particularly in the hippocampus. This dysfunction leads to the abnormal accumulation of misfolded proteins, such as Aβ, which further instigates a vicious cycle of neuroinflammation, synaptic plasticity damage, and mitochondrial dysfunction. Ultimately, these processes result in neuronal dysfunction and cognitive impairment.

While studies on biomarkers related to UPS components have advanced, they continue to encounter challenges, including significant heterogeneity and a lack of sufficient correlation between central and peripheral measurements (Feng et al., 2025; Zhang L. et al., 2024). In terms of treatment strategies, interventions targeting UPS show potential at multiple levels, including enhancing UPS function, clearing protein aggregation, and inhibiting neuroinflammation (Chen et al., 2023; Hu et al., 2022). Future research should focus on clarifying the dynamic changes of UPS across different brain regions through spatiotemporal-specific genetic manipulation. Additionally, it is essential to validate high-sensitivity biomarkers using multi-omics and imaging techniques, as well as to develop targeted drugs capable of penetrating the blood-brain barrier (Ostertag et al., 2024; Patrick et al., 2023). Additionally, precise intervention plans should be developed based on individual patient characteristics, and the synergistic effects of UPS-targeted therapy with anti-inflammatory, antioxidant, and other strategies should be explored.

In conclusion, addressing the dysfunction of the UPS offers a promising new strategy for understanding and treating PND. By conducting continuous in-depth research on the underlying mechanisms, developing biomarkers, and exploring innovative therapies, we aim to reduce the incidence of this serious postoperative complication and enhance the long-term quality of survival for elderly surgical patients.
